# The circadian rhythm: an influential soundtrack in the diabetes story

**DOI:** 10.3389/fendo.2023.1156757

**Published:** 2023-06-27

**Authors:** Amirali Hariri, Mina Mirian, Ali Zarrabi, Mohammad Kohandel, Maryam Amini-Pozveh, Amir Reza Aref, Aliye Tabatabaee, Pranav Kumar Prabhakar, Ponnurengam Malliappan Sivakumar

**Affiliations:** ^1^ Department of Pharmaceutical Biotechnology, School of Pharmacy and Pharmaceutical Sciences, Isfahan University of Medical Sciences, Isfahan, Iran; ^2^ Department of Biomedical Engineering, Faculty of Engineering and Natural Sciences, Istinye University, Istanbul, Türkiye; ^3^ Department of Applied Mathematics, Faculty of Mathematics, University of Waterloo, Waterloo, ON, Canada; ^4^ Department of Prosthodontics Dentistry, Dental Materials Research Center, Dental Research Institute, School of Dentistry, Isfahan University of Medical Sciences, Isfahan, Iran; ^5^ Belfer Center for Applied Cancer Science, Dana Farber Cancer Institute, Boston, MA, United States; ^6^ Translational Sciences, Xsphera Biosciences Inc., Boston, MA, United States; ^7^ School of Pharmacy and Pharmaceutical Sciences, Isfahan University of Medical Sciences, Isfahan, Iran; ^8^ Department of Medical Laboratory Sciences, School of Allied Medical Sciences, Lovely Professional University, Phagwara, Punjab, India; ^9^ Division of Research and Development, Lovely Professional University, Phagwara Punjab, India; ^10^ Institute of Research and Development, Duy Tan University, Da Nang, Vietnam; ^11^ School of Medicine and Pharmacy, Duy Tan University, Da Nang, Vietnam

**Keywords:** diabetes mellitus, circadian rhythm, chronotherapy, metabolic syndrome, sleep disorder, lifestyle

## Abstract

Type 2 Diabetes Mellitus (T2DM) has been the main category of metabolic diseases in recent years due to changes in lifestyle and environmental conditions such as diet and physical activity. On the other hand, the circadian rhythm is one of the most significant biological pathways in humans and other mammals, which is affected by light, sleep, and human activity. However, this cycle is controlled via complicated cellular pathways with feedback loops. It is widely known that changes in the circadian rhythm can alter some metabolic pathways of body cells and could affect the treatment process, particularly for metabolic diseases like T2DM. The aim of this study is to explore the importance of the circadian rhythm in the occurrence of T2DM via reviewing the metabolic pathways involved, their relationship with the circadian rhythm from two perspectives, lifestyle and molecular pathways, and their effect on T2DM pathophysiology. These impacts have been demonstrated in a variety of studies and led to the development of approaches such as time-restricted feeding, chronotherapy (time-specific therapies), and circadian molecule stabilizers.

## 1 Introduction

According to the World Health Organization (WHO), diabetes mellitus (DM) is a metabolic disorder characterized by high level of blood glucose (1) that could damage most of the vital organs including the cardiovascular system (2), eyes (3), kidneys (4), and nervous system (5, 6). More than 90% of all instances of diabetes mellitus are type 2 diabetes mellitus (T2DM) (7), which is characterized by a combination of two different factors: inability of the pancreatic β-cells in producing sufficient amounts of insulin and lack of insulin adsorption by its targeted cells (8). Type 2 Diabetes Mellitus patients are normally obese people with accumulation of body fat, especially in the abdominal area (9). The worldwide changes in the lifestyle patterns including consumption of high-calorie foods (10), and the aging population (11) are the primary causes of the T2DM epidemic.

Fine-tuning the molecular pathways involved in the synthesis and release of insulin and their response in tissues is required to ensure that insulin would meet the body’s metabolic demands (12). Therefore, the smallest metabolic imbalance in any of the relevant pathways could lead to the pathogenesis of T2DM (13). For instance, Adipokine dysregulation and increased free fatty acid release from adipose tissue are two of the inflammatory events that contribute to the development of insulin resistance in the whole body (14).

According to the epidemiological statistics, the predicted rates of T2DM are alarmingly high. The International Diabetes Federation estimates that by 2045, there will be 700 million people worldwide with diabetes (7). In 2019, 4.2 million deaths occurred among people with diabetes between the ages of 20-79 years old. Moreover, in the same year, more than $720 billion was paid for the treatments related to the diabetes, showing its extreme economic burden. The true burden of disease is likely underreported as one in three people with diabetes is underdiagnosed. Most patients with diabetes are middle-aged (40-59 years old) (15) and more than 80% of them live in low- to middle-income countries, which presents additional treatment challenges. Cardiovascular disease (CVD) is the main cause of mortality in T2DM patients compared to those without diabetes, while it could also lead to other vascular-related outcomes and stroke (2). Besides, diabetes can affect other organs such as pancreas (both β and α cells) (16), liver (17), muscle (18), kidneys (19), brain (20), gut (21), and adipose tissue (22). Furthermore, changes in gut microbiota (23), immunological diseases (24), and inflammation (25) are increasingly being recognized as important pathophysiological variables in the onset of metabolic syndrome (26).

Type 2 diabetes can result from the interaction of numerous inherited and environmental risk factors (27, 28). One of the interesting factors that has been proven to have a role in prevalence of T2DM is genetic and environmental changes in circadian rhythm. For example, T2DM is significantly more common in those who work in shifts, such as night and evening shifts (29). It has been demonstrated that days and/or weeks- long exposure to acute circadian disruption leads to changes in glucose metabolism as well (30).

## 2 Pathogenesis of T2DM

Pathogenesis of T2DM is fueled by inducing insulin resistance in skeletal muscle, liver, and fat so that insulin signaling is disturbed (31). Insulin resistance leads to a significant reduction in the glucose clearance, especially through the skeletal muscles that are the primary organ responsible for the postprandial glucose reduction (32). Muscle insulin resistance is due to a combination of factors, including but not limited to 1) insufficient insulin-mediated transport of glucose transporter type 4 (GLUT4) to the plasma membrane; 2) reduced glycogen accumulation; 3) decreased oxidation of glucose; and 4) altered the activity of mitochondria (32, 33). Fasting hepatic glucose production is elevated in an insulin resistance caused by impaired regulation of gluconeogenesis. In a hepatic insulin resistance state, livers produce more glucose after a meal since their ability to metabolize glucose is impaired (34). Finally, reducing the insulin-mediated glucose transport, lipid absorption, lipolysis, and inflammation in adipose tissue can lead to an increase in the plasma free fatty acids (FFAs) (35, 36).

Cellular insulin resistance is resulted from the accumulation of abnormal lipids in insulin-sensitive tissues like liver, muscles, and adipose tissue (36). Indeed, activation of insulin signaling cascade in muscle and liver cells is inhibited by the accumulation and trafficking of lipid messengers such as ceramides and diacylglycerols that are elevated in obesity (37). Besides, cumulation of intracellular diacylglycerols and ceramides could induce the inactivation of insulin receptor substrates 1 and 2 (IRS-1 and IRS-2) and create insulin resistance (38, 39). Protein kinase C and other atypical serine/threonine kinases trigger this pathway (40). Poor adipocyte metabolism has been linked to both obesity and T2DM, leading to an increase in lipolysis and FFA levels and an elevation in the expression level of inflammatory cytokines such as tumor necrosis factor-α (TNF-α) and interleukin-6 (IL-6) from activated adipose tissue macrophages (41–43). As a result, abnormal metabolism in adipose tissue of patients with T2DM is attributed to the excessive fat storage in target tissues via mediating alterations in the insulin signaling cascade in the skeletal muscle and liver.

Hyperglycemia cannot occur without the presence of both insulin resistance and pancreatic islet failure, both of which are characteristic of T2DM (44). Patients with T2DM who suffer from islet failure have impaired glucose-stimulated insulin production and uncontrolled glucagon release. Reduced β-cell populations and impaired β-cell secretory capacity contribute to the impaired glucose-stimulated insulin secretion (45). Increased apoptosis of β-cells along with the development of dedifferentiated α-cells have been linked to the β-cell mass reduction observed in type 2 diabetes (45). Moreover, exposure to islet amyloid peptide oligomers (IAPP), hyperlipidemia, and hyperglycemia is associated with the activation of intracellular oxidative and/or endoplasmic reticulum stress (46, 47). There is also limited evidence that changes in β-cells number and activity is the underlying reason of the defected glucagon suppression observed in diabetes; however, these possibilities need further investigation (48, 49).

### 2.1 Glucose homeostasis mechanism

Signals from organs such as pancreas, brain, gut, and liver exert considerable control on blood glucose homeostasis. The rise in blood glucose levels following a meal is the primary stimulus for insulin release from β-cells (50–52). The glucose transporter 2 (GLUT2) facilitative glucose transporter, found on the surface of β-cells, is responsible for glucose uptake from the circulation (53). During glycolysis, as glucose enters the cells and its level raises, the ATP/ADP ratio and ATP production increase (54). The increased ATP leads to the inhibition of ATP-sensitive K^+^ channels (K_ATP_), channels that are responsible for the constant resting potential in the absence of stimuli via pumping K^+^ ions (55, 56). This leads to the reduced outward K^+^ current, followed by membrane depolarization, and thus, opening the voltage-gated Ca^+^ channels (VDCC) (57, 58). Insulin is secreted when granules containing the hormone fuse with the cell membrane, a process triggered by the increased intracellular calcium concentration.

The action of insulin on cells is mediated by its attachment to the extracellular subunits of insulin receptors on the cell surface; that leads to the phosphorylation of this receptor, the tyrosine receptor kinases (RTKs), followed by the activation of downstream pathways mediating the cellular effects (59, 60).

Another hormone emitted by the pancreas is called glucagon, which is released by alpha cells in the pancreatic islets when blood glucose levels drop. It counteracts with the insulin role in glucose regulation via increasing the production and secretion of glucose. Ketogenesis and lipolysis in the liver are both boosted by glucagon (51). The liver senses glucose levels in the portal vein and sends a signal to the arcuate nucleus (ARC) via vagal afferents, leading to smaller meals and increased postprandial satiety (61, 62). In addition to activating the G-protein-coupled receptors (GPCR) pathways in the ARC, glucagon has also the ability to pass through the blood-brain barrier (BBB) in animal models which suggests that it may directly act on the central nervous system to control hunger (63, 64).

Amylin, which is released together with insulin by pancreatic cells, inhibits the activity of orexigenic neurons in the anterior cingulate cortex to reduce hunger (65). Amylin inhibits gastric secretions and postprandial glucagon release through stimulating the region postrema in the medulla of the brainstem. Somatostatin, the hormone secreted from the pancreatic cells, has roles in regulation of appetite, digestion, and glucose metabolism via exocrine, endocrine, and brain pathways (66–68). It could suppress the expression of different hormones, from glucagon and insulin to gastrin, prolactin, secretin, and thyroid stimulating hormone (69), digestive enzymes, stomach acid, and bile produced by the digestive system (70–73).

The adrenal glands produce corticosteroids, which regulate many bodily functions. The release of this class of hormones follows a 24-hour cycle, controlled by the circadian clock. They can be broken down into two primary groups: mineralocorticoids and glucocorticoids (74). Glucocorticoids have anti-inflammatory effects in addition to regulating carbohydrate, protein, and lipid metabolism, whereas mineralocorticoids such as aldosterone manage the balances of fluid and electrolyte through modifying the renal tubules activity (74). Cortisol, the primary endogenous glucocorticoid in human physiology that is related to the stress, stimulates gluconeogenesis to raise blood glucose levels under both stress and hypoglycemia (75, 76).

**Figure 1:**
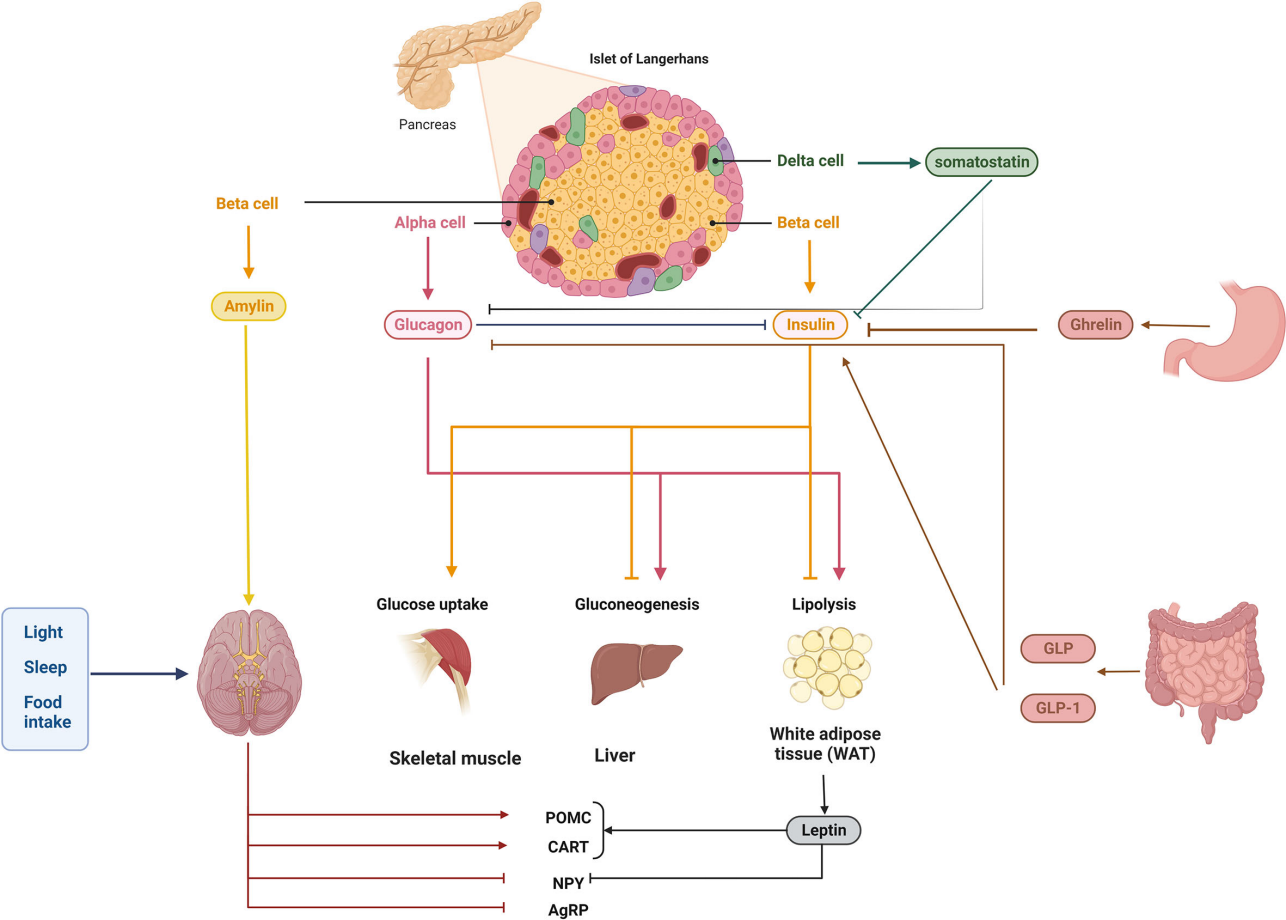
Homeostasis of blood glucose is controlled by a complex network of interactions. Multiple signals from the pancreas and further away from the brain, liver, muscle, intestine, stomach, and adipose tissue regulate blood glucose homeostasis. Glucagon, insulin (together with amylin), pancreatic polypeptide, and somatostatin are produced by cells in the pancreatic islets. Islet cells emit insulin in response to high glucose, such as from food intake; this insulin is detected by numerous peripheral tissues, which then synthesize or induce several molecules/pathways, such as lipogenesis and gluconeogenesis, and block others, such as glycogenolysis. Importantly, insulin secretion can be regulated both locally and at a distance, for example, by hormones and incretins in the colon and the stomach. Blood glucose homeostasis can be regulated by endogenous hormones like glucocorticoids, which also display rhythmicity, and by exogenous variables like light exposure and food consumption, which modulate circadian rhythms.

Postprandially, cells in the digestive tract secrete a class of peptide hormones called incretins, which play a role in controlling both blood glucose and nutrient absorption. Gastric inhibitory peptide (GIP) and Glucagon-like peptide-1 (GLP-1) are two primary incretins that lower blood glucose via increasing the insulin release from pancreatic cells (77, 78). In addition to controlling the adsorption rate of nutrients into the body, incretins reduce the speed of stomach emptying. Dipeptidyl peptidase-4 (DPP-4) could inhibit the GLP-1 and GIP activity, so the inhibitors of DPP-4 along with several types of GLP-1 and GIP analogues have been applied in clinical therapy of T2DM; however, more research is needed to clarify the therapeutic use of these medications for T2DM (79) (**Figure 1**).

### 2.2 Circadian rhythm of glucose homeostasis: Diabetic vs healthy

It has been known for nearly half a century that normal human subjects exhibit a diurnal cycle in glucose tolerance. The rise in plasma glucose concentrations is much lower in the evening than in the morning after ingesting glucose orally or receiving an intravenous infusion of glucose. Mice and rats, like humans, have been shown to have diurnal rhythms (80). They have a lower glucose tolerance during the day (resting phase) than at night since they are nocturnal (active at night). The reasons behind impaired glucose tolerance include insulin resistance, high hepatic glucose production (HGP) from the liver, and impaired β-cell function. Research shows that insulin sensitivity and β-cell responsiveness to glucose are both lower in healthy adults after dinner than before breakfast. In people with normal glucose levels, the function of HGP in the diurnal regulation of glucose homeostasis is unclear. Researchers have found conflicting results regarding the diurnal variation in HGP: some have found a decrease in HGP associated with sleep and an increase in HGP at dawn, while others have found no diurnal rhythm in 24-hour fasting HGP or a decrease in HGP before breakfast compared to the lunch and dinner. One possible explanation for the divergent findings is that the participants in these trials had diverse eating habits on the day of testing. Patients with T2DM commonly experience the dawn phenomenon (hyperglycemia in the morning), which may be explained by the diurnal cycle of HGP. The increased HGP in the morning before breakfast has been linked to both a circadian modulation of HGP, as demonstrated by rodent research, and a prolonged overnight fast, which causes a rush of counterregulatory hormones (such as cortisol, growth hormones, and norepinephrine) (81–83).

Patients with T2DM frequently have fasting/morning hyperglycemia, which can be partially attributed to an increase in the overnight production of endogenous glucose. Endless laboratory work has been prompted by the observation of the daily fluctuations of glucose regulation in an effort to pinpoint and characterize the underlying endogenous circadian rhythms of glucose regulation. With the need to better understand diabetes and its effects, more studies in differential glycemic control over the course of the day in people with and without diabetes is needed (84).

## 3 The circadian rhythm: an overview

Without any environmental cues, circadian rhythms are generated by an internal molecular clock that runs for about 24 hours. A “master” clock present in the suprachiasmatic nucleus (SCN) of the hypothalamus coordinates the molecular clocks in several peripheral tissues into a unified, hierarchical system. About 20,000 neurons make up the SCN to create a highly integrated circadian network (85). Light signals from the retina are processed by the master clock, which then relays the obtained information to the peripheral clocks through the endocrine and systemic cues (86, 87). The molecular architecture and the ability to generate sustained circadian rhythms are shared by the master and peripheral clocks situated in SCN neurons and across peripheral tissues, with the degree of intercellular connectivity between these clocks being the primary distinction.

**Figure 2:**
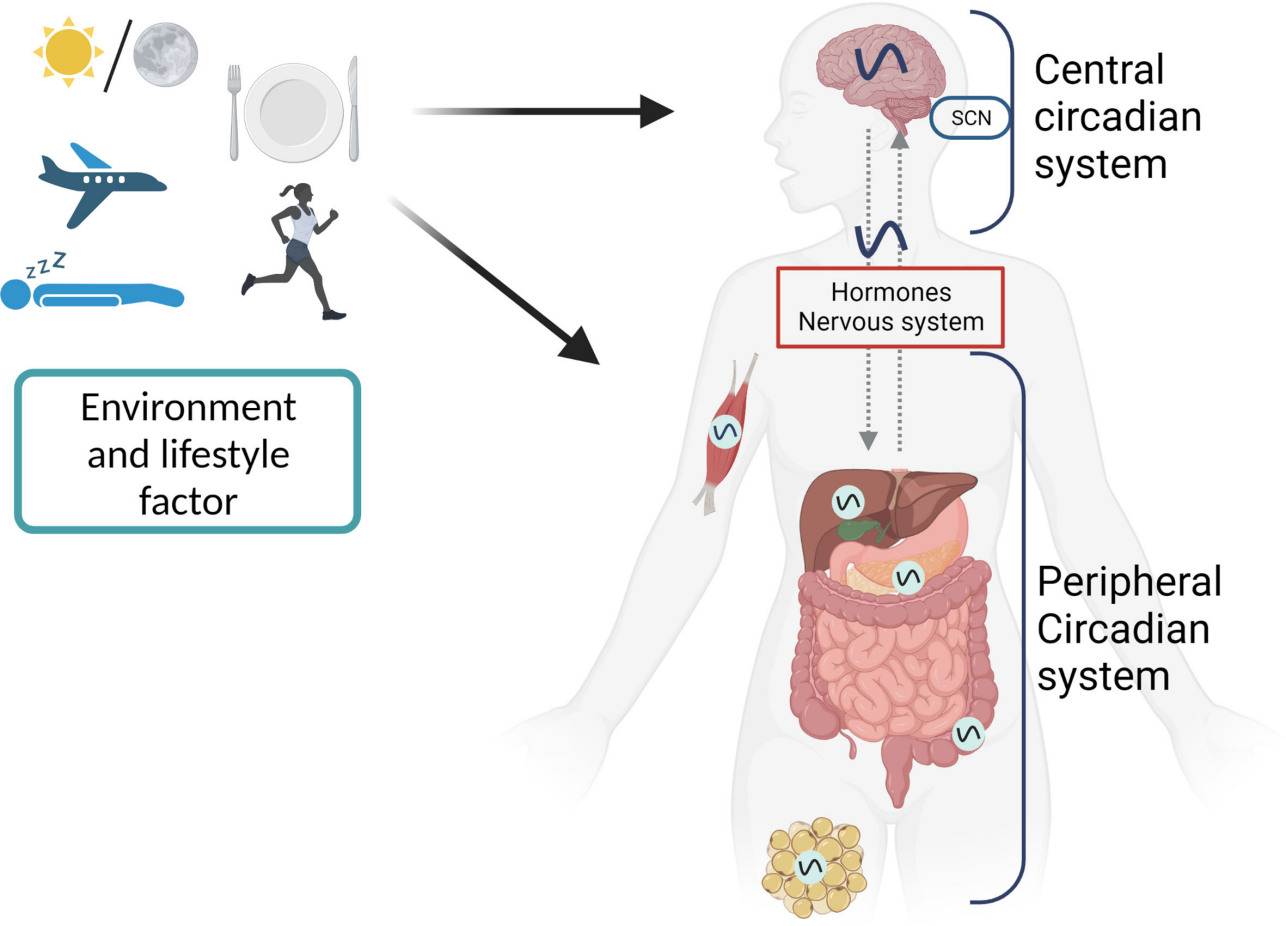
The first level of circadian disruption occurs when environmental cycles and/or behavioral cycles are out of sync with the SCN. Second, internal misalignment between the central clock and the peripheral clocks, generated by, for example, misaligned meals, can create circadian disruption at the organismal level. Another manifestation of this is an improper phase relationship between the peripheral clocks of various organs. Desynchronization between cells inside tissue can alter circadian rhythms on a cellular level.

The systemic changes (like body temperature), metabolic changes (for example by changes in exercise and food intake), changes in sleep pattern (e.g. shift work or jetlag) and the circulating hormones are the regulators of the phase of the SCN clock, while the phase of peripheral clocks can be adjusted independent of the SCN clock (85, 86, 88). This is related to the high degree of intercellular coupling of the SCN neurons, which create a neuronal network resistant to the phase perturbations of the internal cues. This networking logic makes sure that the master clock always stays on time by keeping its intrinsic 24-hour timing in sync with the solar cycle, while the peripheral clocks adjust to show the local metabolic status of the tissues in which they function (**Figure 2**) (85).

Pancreatic islet cell subtypes, skeletal myocytes, adipocytes, and hepatocytes are all examples of metabolically important peripheral tissues/cells that have self-sustaining and cell-autonomous circadian oscillators (89–93). Without consistent stimulation from the SCN, these cell-autonomous oscillators can keep cellular processes rhythmic over the course of a 24-hour day (94). However, the SCN clock appears to integrate a complicated multi-level hierarchical oscillator network by coordinating and synchronizing tissue circadian oscillators in response to neuronal, humoral, and behavioral inputs (95, 96).

It was previously believed that the SCN only used a single biological mechanism for the regulation of the phase entrainment and circadian physiology of the peripheral tissue oscillators; however, recent research suggests otherwise (97). The autonomic nervous system, specifically the parasympathetic and sympathetic axons, aids in SCN-dependent entrainment of circadian rhythms. Autonomic regulation of the hepatic circadian clock, which is responsible for the circadian rhythms in the production of hepatic glucose and ambient glycemia, is mediated by the sleep-wake circadian network (98, 99). The SCN plays a pivotal role in cell types that are vulnerable to daily dietary challenges by indirectly managing the entrainment of peripheral oscillators via modification of the fasting/feeding cycles. It is suspected that a combination of food-related metabolites and gut-related hormonal factors mediates the circadian entrainment of peripheral oscillators in response to meals, although the specific processes involved remain unknown (100). The SCN also controls circadian cycles in temperature and hormones (e.g., glucocorticoids) (101). In mammals it manages peripheral oscillator entrainment and uses redundant communication channels to fine-tune the temporal regulation of a wide variety of circadian rhythms.

### 3.1 Molecular pathway of the circadian rhythm

**Figure 3:**
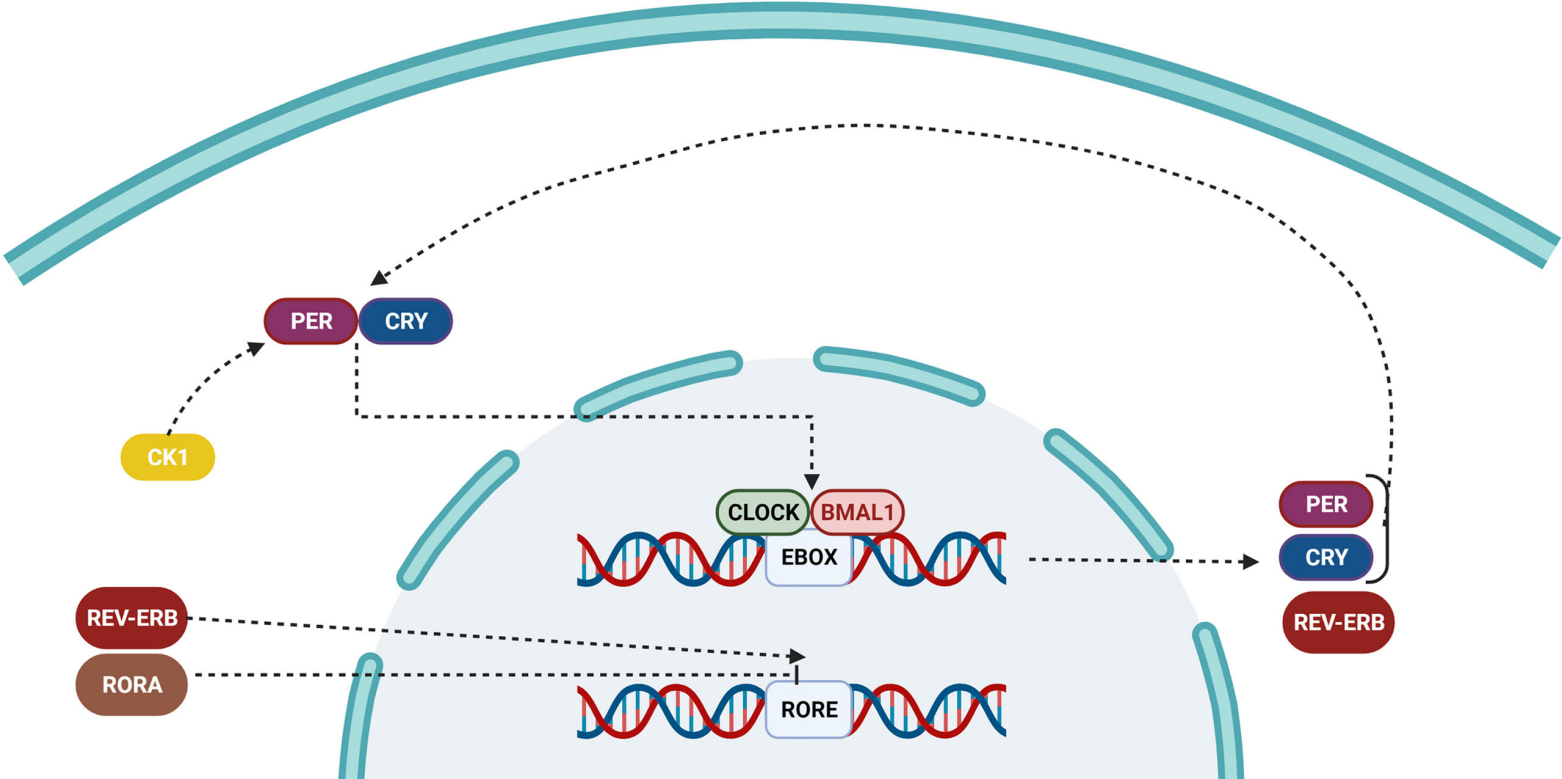
Pathway of the circadian clock inside a cell. Over time, the accumulation of PER and CRY proteins binds to CLOCK/BMAL1, and switches it from an active to an inactive state, preventing it from repressing the transcription of downstream genes. The ROR/REV-ERB complex controls the primary feedback loop by influencing RORE.

The cell-autonomous molecular clock of the mammals consists of two transcription/translation feedback loops (TTFL) that interact with each other and generate strong 24-hour rhythms for gene expression. The core of TTFL is composed of four integral clock proteins: two activators (CLOCK and BMAL1) and two repressors (Period circadian protein homolog;PER1 and PER2) (102, 103). Their location and stability are regulated by kinases and phosphatases (kinases: CKI, phosphatases: PP1, PP5). CLOCK: BMAL1 is a type of transcription factor with basic helix-loop-helix-PAS (PER-ARNT-SIM) structure that stimulates the expression of the *Per* and *Cry* (Cryptochrome) repressor genes and other genes responsible for the clock-controlled output (104). The heterodimer formed in the cytoplasm between PER and CRY proteins is transported to the nucleus, so that it could inhibit further transcriptional activity via interacting by CLOCK: BMAL1. After about 24 hours, PER and CRY proteins are destructed through a ubiquitin-dependent processes, releasing the regulation of CLOCK: BMAL1. Phosphatases (PP1 and PP5) are used for preventing and regulating the activity of casein kinases (CKI and CKI, respectively), which is a critical factor for controlling the clock intrinsic period (**Figure 3**) (105, 106).

Sleep phase issues are shared by mice and humans when either the intrinsic duration of the clock is reduced (S662G) or a loss-of-function mutation occurs in CKI (T44A). By altering the location and stability of PER, pharmacological inhibition of casein kinases was shown to have a significant role in period length determination. Transcriptional activators (retinoid-related orphan receptors RORa, b, and c) and repressors (REV-ERBa and REV-ERBb) are involved in generation of the second TTFL (107, 108). ROR/REV-TTFL-induced delay in the expression of *Cry1* is critical for the appropriate circadian timing; however, rhythmic changes in the abundance of BMAL1 are not necessary for driving the core loop of TTFL. Gene expression is appropriately timed for local physiology thanks to the cooperation and interlocking of feedback loops that induce phase delays in the output of the circadian transcription and make a robustness against the environmental disturbance and probable noises (109).

## 4 Diabetes and the circadian rhythm: a new frontier in diabetes research

### 4.1 Genetic signatures in the human genome

The metabolic risk of T2DM can be increased or decreased by specific single nucleotide polymorphisms (SNPs) in humans. Lipid metabolism can also cause SNPs in circadian genes (such as *PER3*), and on the other hand, the presence of SNPs in circadian genes (such as *CLOCK* and *CRY1* in intestinal cholesterol absorption) can play a role in fat metabolism and increase the chance of T2DM (110–114). Risk factors for developing metabolic syndrome (such as hypertension) are also associated to a SNP presented in the structure of neuronal PAS domain protein 2 (NPAS2), a paralog of CLOCK with the ability of binding to the BMAL1 (115). *Bmal1* SNPs are associated with high blood pressure in addition to T2DM, hyperglycemia, and gestational diabetes (116, 117). Patients with *Cry2* and *Per2* SNPs are prone to the glucose tolerance disorder, and individuals with *Per2* SNPs, who overeat and experience stress-related eating, tend to acquire more weight (115, 118, 119).

These SNPs could also affect the expression level of several other genes. For example, the sleep/wake cycles are aided by the secretion of more melatonin in the evening and less during the day. Elevated fasting glucose levels could impair cellular functions and increase the risk of T2DM in the presence of two SNPs in the melatonin receptor 1B gene (*Mtnr1b*) (120, 121). Melatonin secretion patterns could be affected by these SNPs and lead to an increased risk in diabetes. Accordingly, it seems that the risk of metabolic syndrome, obesity, and T2DM could be significantly influenced by SNPs in the molecular circadian clock and the genes regulated by those SNPs (122).

In a 2021 study, researchers showed a genetic network between the circadian cycle and T2DM (123). In this study, integrating gene expression patterns with the biomolecular networks of the genome-scale in diabetes samples allowed for a meta-analysis of data pertaining to genes involved in T2DM and different types of cancers (including bladder, breast, pancreatic, liver, colon, and rectum cancers). They found that both of these disorders shared a common deregulation of a number of genes. For example, although the expression level of *Arntl2* and *Agrp* were increased in T2DM and cancer samples, *Usp2*, *Ezh2*, *Igf1*, *Klf10*, and *Ntrk3* were downregulated (123). Phenotypic changes in T2DM appear to be linked with the malignant transformation, which may partially be related to the alterations in mRNA. Using these genes for survival analysis, they discovered associations between only *Arntl2*, *Usp2*, and *Igf1*. Survival was poor in BLCA and BRCA cancer samples where *Arntl2* was upregulated, while downregulation of *Usp2* and *Igf1* had a negative effect (123).

### 4.2 Peripheral circadian rhythm: a central role in diabetes

#### 4.2.1 Pancreatic circadian rhythm

It has long been understood that individuals’ ability to maintain their blood glucose levels stable throughout the day is varied with the time. Indeed, improved glucose tolerance is related to higher effects of glucose and β-cell targeting secretagogues on insulin secretion, which is first observed at the start of the active circadian cycle (124, 125). Classic experiments using a 72h glucose clamp methodology corroborated these first data, showing that humans exhibit strong circadian rhythms of insulin secretion independent of feeding and glycemia (29). Moreover, the cell-autonomous circadian clock appears to oversee the diurnal rhythms in the glucose-induced insulin secretion. These diurnal rhythms are maintained in the isolated pancreatic islets and pure β-cell populations in human, animal, or *in vitro* models (29, 126). Besides, circadian fluctuations have also been seen in the glucagon production *in vivo* and *in vitro* (islet cultures and isolated α-cells). However, our knowledge of the circadian regulation of the α-cell glucagon response is considerably limited (127).

Many research groups have established the effects and roles of the autonomous circadian clock (e.g., Per1 and Per2) in pancreatic islets (127–130). The results of these studies demonstrated that clock gene expression in islet cells exhibits robust independent circadian rhythms with a clear 24h period, phase, and amplitude of circadian oscillations. Moreover, they showed that SCN entrains the circadian clocks phases of the islets via modulating the feeding behavior. It was also seen that shifts in photoperiod and/or obesogenic meals can jumble the islet circadian clock (131). Besides, the pure populations of α- and β-cells exhibit robust cell-autonomous circadian oscillators, with discrete phases in transcriptional and functional oscillations (89, 132).

Similar to other metabolically active cells such as skeletal muscle and liver, the transcriptional profile of islet cells is subject to circadian oscillations regulated by the cellular clocks. Around one-third of the transcripts in mouse islets show circadian cycles, which is consistent with previous findings. The biological pathways involved in the secretion and exocytosis of insulin (including SNARE interactions, vesicular transport, and exocytosis), mitochondrial function, processing and transporting of protein in endoplasmic reticulum, and cell turnover regulation, are enriched in circadian-expressed transcripts in islet cells (such as DNA damage and repair and DNA replication) (133, 134). Recent research has shown that each of the distinct islet cell subtypes have their own circadian transcripts, with expression peak during the feeding or fasting (activating or inactivating) of the circadian cycle, which corresponds to the glucagon and insulin release peak (130, 135).

#### 4.2.2 Liver circadian rhythms

Liver plays a critical role in orchestrating the glucose homeostasis’s circadian regulation throughout the day. It is the diurnal change in the amount of hepatic glucose synthesis that regulates the circadian variations in blood glucose under fasting conditions (136). Because of this, circadian rhythms in the concentrations of blood glucose are lost if the circadian clock of SCN (or liver-specific) is disrupted (137). The liver also acts in the circadian modulation of postprandial glucose metabolism since the circadian changes in the amounts of glucose production by the liver could affect the diurnal cycles of meal tolerance and insulin sensitivity. In addition, both animal and human models have shown that the rates of gluconeogenesis and glycogenolysis, as well as glycogen storage strongly fluctuate during the day (29, 138).

Hepatocytes exhibit specific diurnal oscillations in gene expression and morphological properties such as volume and size of cells (139–141). Hepatocytes display patterns of circadian expression for a number of metabolic genes, including *Glut2*, *Gck*, *Pepck*, and *Gcgr*. These genes are critical for regulating hepatic glucose production, and lipid oxidation in hepatic cells, respectively. Both nuclear and cytoplasmic proteome of hepatocytes undergoes significant diurnal variation associated with rhythm in cellular metabolism, proliferation, DNA repair, ribosome synthesis, and intracellular protein trafficking. Multiple diurnal rhythms in liver function, as well as the corresponding of diurnal oscillations in the transcriptome and proteome of the liver, appear to be primarily regulated by fasting-feeding cycles. Consequently, while assessing the mechanisms regulating daily circadian rhythm in the liver, it is important to distinguish between clock-dependent and clock-independent factors (such as feeding-driven factors) (142). Besides it as important to add that feeding may be a stronger cue for hepatic circadian rhythm than light-dark cycle as feeding restricted to resting phase inverts metabolic rhythm of liver (143, 144).

Scientists have uncovered some of the processes and targeted molecules related to the clock-controlled transcriptional regulation of hepatocytes by utilizing chromatin immunoprecipitation (ChIP-Seq) and deep sequencing. For instance, Rey and coworkers mapped almost 2,000 BMAL1 binding sites across the entire genome in the liver (145). Additional evidence have shown that hepatic clocks are critical mediators of glucose homeostasis since most of the binding sites were located/mapped in promoter areas of genes related to the metabolism of hepatic glucose (like *Glut2* and *G6pc*) and lipid (such as *Agpat6* and *Dgat2*). This finding is supported by the *in vivo* test observation that showed mice with a liver-specific deletion of *Bmal1* exhibit systemic glycemia and glucose intolerance due to the absence of rhythmic regulation of glucose metabolism related genes (e.g *Glut2*) (146, 147). Newer studies built on these observations, demonstrating that hepatic *Bmal1* is essential for controlling hepatic oxidative capacity through orchestrating intracellular mitochondrial dynamics such as mytophagy and fission (146, 148).

Through the activation of different transcriptional and translational regulators, hepatic clocks coordinate the cellular metabolism regulation via an “indirect” method. By performing a ChIP-Seq analysis on binding sites of BMAL1 of the liver, they were able to show that “transcriptional control” was the most significantly enriched annotated functional cluster, included about 82 DNA transcription factors and 18 liver-expressed nuclear receptors. In lipid and cholesterol metabolism, hepatic clocks activate Kruppel-like 10 (*Klf10*) and the nuclear receptor *Ppar* (peroxisome proliferator-activated receptor) (149–151). The fact that both *Klf10* and *Ppar* regulate Bmal1 promoter activity demonstrates the intricate relationship between the circadian clock and metabolism of liver (149, 150). Finally, research have shown that proteins involved in the circadian clock (such as CRY 1 and 2, and PER2) attune the regulation of circadian over hepatic glucose metabolism via modulating the signaling of cAMP and the post-translational function of nuclear receptor. Diabetic mice with overexpression of *Cry1* in the liver had reduced gluconeogenesis and blood glucose levels. Researchers convincingly showed that *Cry1* expression during the circadian clock inhibits the key enzymes of gluconeogenic activation through binding and blocking the Gs subunit of hepatic glucagon receptor (152, 153). Growing documents indicate that hepatic clocks play a critical role in the regulation of glucose production and uptake and insulin sensitivity in T2DM.

#### 4.2.3 Muscle circadian rhythms

It was revealed by *in vivo* and *in vitro* studies skeletal myocytes contain functional, self-sustaining, and independently oscillating circadian rhythm. It is important to note that circadian fluctuations occur for almost every physiological factor involved in glucose homeostasis in skeletal muscle (92, 154, 155). Changes in protein and lipid metabolism, as well as glucose oxidation and uptake, deposition of glycogen, the oxidative capability of mitochondria, and expression and translocation of the GLUT4, occur in skeletal muscle on a daily basis (156–158). Consistent with its basic function, the circadian system increases metabolic flexibility, as evidenced by the fact that the maximum physiological activity of skeletal muscle in uptake and oxidation of glucose is coincided with the commencement of circadian cycles in both human and animal (mouse) tests. In accordance with the transcriptional control by the endogenous circadian clock mechanism, circadian rhythms in skeletal muscle activities (such as glucose absorption) are maintained in isolated muscle cells (158). In addition, T2DM is linked to alterations in the circadian modulation of glucose uptake and oxidation in muscle, suggesting that the disruption of the circadian clock is a factor that could affect the pathogenesis of insulin resistance in skeletal muscle of patients with diabetes (159, 160).

The strongest evidence that confirmed the critical role of biological clock in regulating the function of skeletal muscle is provided by the studies done on *Bmal1* knockout mice models. The loss of *Bmal1* is unique among clock genes because it eliminates circadian rhythms in behavior, physiology, and molecular processes. Notably, muscle-specific transgenic rescue can correct the profound decrease in the mass, function, and mitochondrial density of skeletal muscle resulted from a systemic loss of *Bmal1* (161). *Bmal1* knockout mice models have been established to clarify the function of this gene in the regulation of glucose metabolism in skeletal muscle. It is interesting to note that Bmal1 knockouts generally show a significant reduction in glucose and insulin tolerance, resulting from reduced insulin-stimulated glucose uptake, which is a major factor in T2DM (162, 163).

Disturbing the glucose uptake related to the *Bmal1* deletion could also reduce the expression level of TBC1D1, a protein essential for GLUT4 translocation from the cytoplasm to the plasma membrane (164, 165). Notably, reduction in the amounts of glucose transport documented in patients with T2DM also affects skeletal muscle. Deletion of *Bmal1* in muscle could change the cellular metabolism toward increasing the lipid usage and storage, and reduce the expression and activity of essential metabolic enzymes that are responsible in the breakdown and oxidation of glucose (164). Again, this is consistent with the phenotypic alterations in T2DM patients’ skeletal muscles. Extensive experimental data suggest that impaired insulin-mediated glucose transport, absorption, storage, and oxidation in skeletal muscle contribute to the onset and progression of T2DM (166).

#### 4.2.4 Adipocyte circadian rhythm

In addition to act as an energy source, white adipose tissue (WAT) has metabolic and endocrine functions including blood glucose level regulation. The body utilizes the triglycerides (TGs) stored in WAT for energy through a process called lipolysis, in which the TGs are broken down into FFAs and glycerol (167). Lipolysis and lipogenesis, as two main paths of fat metabolism in the body, play a very important role in glucose homeostasis, since the excess fat are accumulated in different tissues especially liver and muscles, and cause insulin resistance and interfere with glucose homeostasis (168). Human plasma-free fatty acid concentrations have been shown to vary throughout the day, and this diurnal variation has been believed to be caused by the cyclic changes of the insulin signaling cascade activation in adipose tissue (169).

The circadian clock has been demonstrated to govern adipose tissue functions such as lipolysis, adipogenesis, and metabolic inflammation in mammals because of the dramatic daily variations in energy needs (170, 171). Obesity is reliably induced in Clock and/or Bmal1 mutant mice, likely as a result of elevated levels of plasma FFAs and triglycerides. Studies in rodents and humans have also uncovered the importance of circadian expression for many of the genes that are critical for the appropriate regulation of metabolism of adipocyte in different adipose tissue depots, suggesting that circadian clocks play a role in regulating adipocyte metabolism (91, 172). As an illustration, one study demonstrated that the heterodimer of BMAL1 and CLOCK had a transcriptional regulation on the activity of two important lipolysis pacemaker enzymes that lead to the clock-regulated release of FFA and glycerol (173). Adipocytes had a reduced ability to produce polyunsaturated fatty acids in Bmal1-deficient animals. This could be due to decreased expression of Elovl6 and Scd1, two critical genes involved in the elongation and desaturation of FFA (173, 174).

Adipose tissue is known as the energy stores as well as one of the largest endocrine organs due to its seemingly endless capacity to expand and produce a wide range of hormones and cytokines. Leptin, adiponectin, resistin, visfatin, omentin, and inflammatory cytokines (such as tumor necrosis factor-alpha (TNF-α), interleukin-6 (IL-6), and monocyte chemoattractant protein-1 (MCP-1)) are just some of the known hormones that are released by adipocytes in both obesity and diabetes (175, 176). Macrophages found in adipose tissue are one of the best-known contributors in the insulin resistance induced by obesity. Adipose tissue macrophages (ATMs) have been demonstrated to convert from anti-inflammatory M2 phase to pro-inflammatory M1 type in diabetes conditions, and release the pro-inflammatory cytokines such as TNF-α, IL-6) and IL-12 (43, 177, 178). Although the precise role of the circadian clock in regulating inflammation in adipose tissue remains unclear, there is mounting evidence that suggests disruptions in these pathways can trigger inflammatory reactions (179). For instance, the expression level of TNF-α and macrophage-1 antigen (MAC1) in WAT was increased in obese mice with a disrupted circadian clock (177, 178, 180). Macrophages with a disturbed circadian clock (*Per2* mutant) showed higher M1 macrophage polarization in WAT and exhibited heightened pro-inflammatory activation in response to LPS. Downregulation of PPAR, an important clock-regulated transcription factor used for the polarization of M2 macrophage, in macrophages replicated the same results in obese mice with *Per1/2*-disrupted myeloid cells (41). Overwhelming evidence suggests that the circadian clock could regulate the biology of adipose tissue from multiple aspects (such as lipogenesis, lipolysis, insulin sensitivity, inflammatory pathways, etc.) so that the disruption of this clock could lead to an increase in the adiposity, insulin resistance, and susceptibility to the development of T2DM (176, 181).

### 4.3 Environmental and epidemiological factors

#### 4.3.1 Light and circadian rhythm

**Figure 4:**
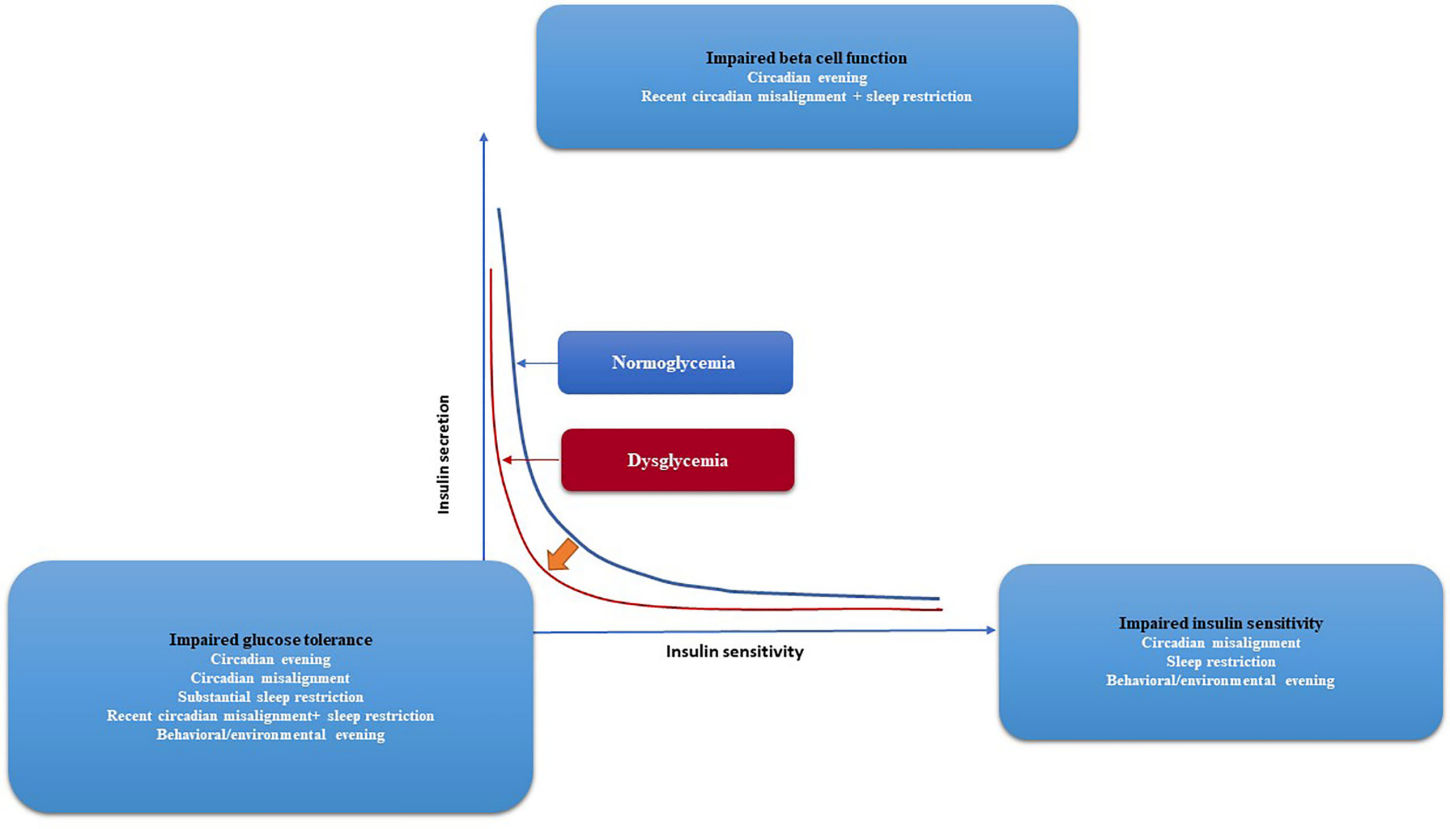
Glycemic control and the risk of developing diabetes, together with the effects of circadian and behavioral factors are depicted graphically. The two hyperbolae for normoglycaemia (blue line) and dysglycaemia (red line) illustrate the correlation between insulin secretion and insulin sensitivity (red line). Lower insulin sensitivity requires more insulin secretion for normal blood glocuse levels. Impaired glucose tolerance arises if insulin secretion is unable to compensate for diminished insulin sensitivity, as shown by a leftward shift of the curve. Circadian disarray, sleep restriction, and, to a lesser extent, behavioral/environmental lateness all have negative effects on insulin sensitivity, according to experiments. Both recent circadian misalignment and nocturnal awakening have negative effects on beta cell function. Evidence suggests that a number of factors—including behavioral/environmental evening, circadian misalignment, significant sleep restriction, recent circadian misalignment with sleep restriction, and severe sleep restriction—combine to reduce glucose tolerance. The progression from normoglycemia to dysglycemia and eventually diabetes may be influenced by circadian and behavioral factors that affect glycaemic regulation.

Constant light exposure decreased the domain (the difference between the highest and lowest points) of the SCN rhythmicity in mice, as measured by electrophysiological monitoring. As a result, food consumption rose while energy expenditure fell (87, 182). In a study in mouse models, the effect of the light cycle on fat and glucose metabolism was investigated. Results demonstrated the effect of light on obesity and development of diabetes. Body weight gain, adipocyte area, glucose intolerance, and insulin resistance were all augmented in low-fat diet (LFD) and high-fat diet (HFD) mice exposed to the circadian-disrupting (CD) cycle under higher intensity (HI) but not lower intensities (LI) (183). In addition, LFD-HI therapy was associated with an increase in blood and hepatic triglyceride levels independent of the light cycle. Another benefit of CD cycle was reflected in the improvement of glucose and lipid metabolism in HFD-LI. Moreover, the deleterious effects of CD cycle on the metabolism were mitigated under the LI condition, particularly in HFD mice (183) (**Figure 4**).

#### 4.3.2 Food intake and circadian rhythm

The hypothalamus, adipose, and hepatic clocks are all affected by a high-fat diet in animal models. Circadian rhythms are also affected by the timing of meals. The postprandial glucose response to a meal may be affected by the time of day, which would have a profound impact on T2DM (184). Earlier mealtime consumption has been linked to lower levels of postprandial glucose in both observational and experimental investigations. Increasing the protein and fat content of evening meals has been found to be a straightforward method for lowering postprandial glucose levels (184). Foods with a low glycemic index (GI) have a negative impact on the glycemic response if they were taken in the morning than later (185). The glycaemic response can be lowered by carefully timing the consumption of fat and protein with carbohydrate items like bread and rice. There is interesting evidence that changing dietary habits such as time and number of meals, the interval between meals, and the amount of each meal can influence postmeal glucose levels and metabolism. Instead of solely considering the dietary content of a meal, people with diabetes can benefit from considering these recommendations to improve their glycaemic management (185).

The majority of food consumed by mice maintained on a 12h light/12h dark cycle (12:12h LD) is consumed during the dark phase. However, in a study that was conducted to determine the effects of the time of feeding on weight gain, it was concluded that mice that were only fed during the light phase acquired more weight compared to those that were only fed during the dark phase (186). In another experiment, the effect of time-restricted feeding (TRF) on preventing obesity and metabolic syndrome was investigated in the liver of *Rev-erb α/β* and *Bmal1* knockout mice and all organs of Cry1;Cry2 knockout mice (187). When these mice were given free access to food, they quickly became obese and displayed metabolic abnormalities that were determined by their genetic makeup. However, when they were fed with the same diet but with TRF, they were in a healthy weight range with no metabolic disorders. It was discovered through transcriptome and metabolome analysis that TRF lowered hepatic fat buildup and improved cellular resistance to metabolic stress (187).

Same as what was seen in animal studies, the timing of meals has been proven to affect the risk of diabetes development in humans. A study in 2018 has shown that there is a direct link between eating dinner late and gene MTNR1B (melatonin receptor 1B), which affects glucose metabolism, increased body fat and weight gain (188). Previously, the relationship of this gene with type 2 diabetes has been proven (189). Since exogenous melatonin has also been demonstrated to cause decreased glucose tolerance, the suggested role of melatonin in this pathway is in line with that discovery (190). Time-restricted eating (TRE), eating within a specific daily window, has been shown to improve the metabolic indices such as atherogenic lipids, weight, visceral fat, and blood pressure in people with metabolic syndrome. In 2019, a randomized controlled clinical trial was conducted to evaluate the association of 9-hour TRF with diabetes risk in men. In this study, 15 men were randomly divided into two early (TRFe) or delayed (TRFd) groups, and during and before (to achieve baseline glucose) the study, the volunteers’ blood glucose and insulin were measured. The level of blood lipids and steroid hormones were measured in all volunteers. The results showed that TRF can improve glycemic responses regardless of whether it is early or delayed (191). The benefits of TRE were tested and reported by a small sample size (n=15–20) and a larger clinical study (192, 193).

#### 4.3.3 Work shift and circadian rhythm

Shift work, and circadian misalignment more generally, are associated with the increased risk of metabolic syndrome, obesity, and T2DM. A real-world investigation comparing day-shift and night-shift workers supports the hypothesis that circadian misalignment is a risk factor for metabolic disorders (134). Results showed that compared to day-shift workers, night-shift workers had higher levels of glucose, insulin, and triacylglycerol after eating a meal. A meta-analysis of 12 observational studies reported that shift work is associated with an increased risk of T2DM (9%), compared to those who have never worked shifts (194). It’s important to note that workers whose shifts alternate throughout the week are at a greater risk than those whose schedules remain stable. This is probably because both food intake and light exposure are an inappropriate time when the body is not prepared for them. Also, similar to the human results, rats subjected by a simulated shift-work procedure (using rotating running wheels) became obese and experienced a flattening of their glucose rhythms. Accelerated cell dysfunction and loss along with impaired GSIS were observed in rats whose 12:12h LD cycles were disrupted by continuous light exposure (131, 195). Research on *Bmal1* knockout mice has revealed the critical function of *Bmal1* in mitigating oxidative stress and adjusting to circadian rhythm disturbances. Therefore, mice with a dysfunctional version of this gene are more likely to develop diabetes (196).

The timing of food consumption, light exposure, and nutrient content have all been recognized as important factors used for regulating the metabolic clock in both rodents and humans. These results suggest that time-specific therapy (chronotherapy) and other strategies that target the circadian system, like the analogs of synthetic circadian protein, could be useful for the management of metabolic syndrome and T2DM.

## 5 Chronotherapy; new weapon in a war against diabetes

Chronotherapy consists of maintaining an optimized circadian rhythm and regulating the timing of drugs to achieve more efficacy and fewer side effects (197). An example of medication timing is prescribing statins to be used at night. The reason is that statins target 3-hydroxy-3-methylglutaryl coenzyme A (HMG CoA) reductase. The activity of this enzyme is highest at night and during sleep; therefore, targeting it at night with statins can be more effective (198).

A dopamine agonist, bromocriptine, is an adjunctive therapy for T2DM. Following a 24h cycle, hypothalamic dopaminergic activity drives hepatic gluconeogenesis and adipocyte lipolysis (199). When taken within 2h of awakening, bromocriptine is expected to inhibit this drive, helping to prevent hyperglycemia and dyslipidemia (199). Despite FDA approval for the treatment of T2DM and evidence of the drug’s effectiveness in lowering blood glucose levels, bromocriptine is rarely used in actual clinical practice (199). In a study in 2021, results showed that in addition to greater effectiveness, the reduction of complications, especially cardiovascular complications, can be seen in the treatment with bromocriptine based on the circadian rhythm; however, with all this evidence, further preclinical and clinical studies are still needed to identify the exact relationship between circadian rhythm and bromocriptine (200, 201).

Despite laboratory evidence that showed the time-dependent effects of metformin on blood glucose and its interaction with the circadian rhythm molecular components, no clinical trials have looked at the optimal time of day to take this drug (202, 203). Scientists found that the time of day significantly affected the initial drop in blood glucose and the levels of lactate in the blood of healthy mice after metformin treatment (204). They further showed that circadian time has significant effects on the activation of AMP-activated protein kinase (AMPK) in the liver via affecting the kinetics of metformin. Loss of *Bmal1* expression in the liver modifies the AMPK induction and blood glucose response to metformin, while can’t completely eliminate the diurnal variations. Combined, these findings highlight the complexity with which circadian rhythms influence physiologic responses to metformin (204). In addition to the effect of the circadian cycle on metformin, it has also had very significant effects on modifying the circadian cycle of different tissues (such as liver and muscles) in diabetes (205, 206).

The endocrine cells of pineal glands secrete a hormone called melatonin, which was the first circadian-based medication to be studied for its potential to treat diabetes (207, 208). Since its production and secretion are governed by direct neural inputs from the SCN, it has been argued that the rhythmic secretion of melatonin is a hormonal response to the central circadian clock (209). Indeed, the SCN and peripheral circadian oscillators are directly affected by exogenous melatonin treatment via the ubiquitously expressed melatonin receptors. Thus, it has been proven that supplementing with melatonin at the proper time improves the global circadian rhythms in both mice and humans (209, 210). By improving the insulin signaling cascade of the cells, melatonin treatment in animals has been found to reduce obesity and attenuate insulin resistance of the liver and skeletal muscle. In isolated T2DM islets, it was able to enhance the survival of β-cells and improve the insulin secretion (stimulated by glucose); therefore, reduce the oxidative and endoplasmic reticulum stress (211–213). Although many studies have pointed out the potential therapeutic effects of melatonin in the control and treatment of diabetes, there is still a need to conduct targeted clinical studies for determining the effectiveness of this drug in T2DM treatment (214–217). In another study, the effectiveness of melatonin and metformin at the same time in the treatment of diabetes was discussed in rat that had become diabetic due to circadian rhythm disorder and obesity (218). As a stand-alone therapy, melatonin improved the activity of circadian rhythms, suppressed the induction of β-cell failure, and enhanced the tolerance of glucose. On the other hand, metformin improved the sensitivity of insulin and glucose tolerance without affecting circadian rhythms; however, it was taken to help with sleep issues (218). Importantly, melatonin and metformin demonstrated synergistic activities to ameliorate obesity, insulin sensitivity, circadian activity, and suppression of islet cell failure in CDO rats, and so slowing the onset of metabolic dysfunction. Based on the results of this research, it seems reasonable to consider treating metabolic and circadian dysfunctions together as a means of preventing and treating obesity and T2DM (218).

More research on small-molecule pharmacological enhancers of the circadian system has been conducted in recent years to improve the clock-regulated physiological outputs. Subsequently, several pharmacological agents have been created with established efficacy in modifying metabolic function in pre-clinical investigations, all of which focus on the core circadian oscillator (219, 220). Initially, thiazolidinedione, a medication used to treat diabetes, was identified as the first synthetic agonist for ROR (Retinoic Acid-Related Orphan Receptors) and the only medication available for the treatment of insulin resistance (221, 222). Nobiletin, another ROR inhibitor and a naturally occurring flavonoid has been discovered to increase clock amplitude through activation of the ROR nuclear receptor (223). Specifically, administration of Nobiletin for an extended periods of time improved the glycemia, glucose tolerance, insulin resistance, and ectopic fat formation in two different mouse models of obesity and T2DM (224, 225). Rev-ERB agonist, a recently found pharmacological regulator of the circadian system was shown to have the capability of modifying the gene expression of circadian metabolic and ameliorate glycemia, lipidemia, and ectopic fat deposition in mice (226). One of these compounds is berberine, which intervenes in the circadian cycle through three pathways; 1) reducing the activity of the luciferase reporters Nlrp3 and Bmal1, 2) increasing the repressor activity of REV-ERB, and 3) reducing the expression levels of REV-ERB target genes (227). In another clinical trial about the effect of berberine in the treatment of diabetes, the results provided evidence that berberine is a safe and effective therapeutic agent for diabetes, particularly when used in combination with other medications. This may be useful in the future for directing the clinical application of berberine and the development of drugs to treat type 2 diabetes and dyslipidemia (228).

Cell-based phenotypic screening for drugs that modulated circadian rhythmicity led to the discovery of the first CRY agonist (KL001) (229, 230). The mechanism of action of KL001 involves blocking the interaction of the ubiquitin ligases that bind to the main binding pocket of CRY and tag them for destruction. In order to extend the repressive phase of the clock, CRY’s lifetime can be extended by binding tiny molecules in the binding pocket (229). Improvements in glucose tolerance in the db/db mice model of diabetes have been observed *in vivo* with compounds that were discovered through *in silico* drug design research began with KL001 (231).

## 6 In-silico approach: systematic study in biology and pharmacology

Understanding diseases, determining the relationship between various cellular pathways, and selecting an appropriate treatment all benefit greatly from the use of various computational and simulation technologies (such as artificial intelligence, molecular docking, etc.). Systems biology refers to the discipline that uses computational tools and models of complex biological systems to systematically investigate biological phenomena (232, 233).

These computational analyses and sophisticated models have also found application in the field of pharmacology. Therapeutic efficacy and side effect predictions (including organ toxicity and genetic toxicity) and medication repositioning could be improved with the help of these computational investigations. System pharmacology is the name given to this branch of pharmacology (234, 235).

### 6.1 Drug combination prediction

Because of their potential to boost efficacy or lessen unwanted side effects, combination treatments are increasingly being used in the treatment of a wide range of disorders. In addition, optimizing the inhibition of different cell pathways can enhance the process of treating a disease when several pathways contribute to its incidence or treatment. Given the large number of available medications as well as intricate physiological pathways, the use of computational methods to predict the ideal therapeutic combination is critical and can save time and money on various research (236–238).

For example, in 2021, Teng and et.al, used *In silico* method for predicting effective drug combinations for T2DM treatment. They compiled data on genes linked to T2DM and built a disease module for the condition. They then identified promising medications by assessing their spatial closeness to the disease module. Gene Set Enrichment Analysis (GSEA) was then used to narrow down the list of possible medications based on the drug-induced gene expression profiles. This network-based approach had also been used in predicting potential drug combinations for type 2 diabetes (237).

Considering the relationship between circadian cycle and type 2 diabetes and the specificity of different cellular pathways, the use of these computational methods can speed up the use of combination therapy based on circadian cycle in the treatment of diabetes.

### 6.2 Drug repositioning

To date, more than 5,000 drugs have been approved by the US Food and Drug Administration in various therapeutic indications, and many drugs are in different phases of clinical studies. This volume of drugs and available data on their effects on cells and different cellular pathways gives us the possibility to find new targets for drugs that have previously been approved and used for other targets without the need to design new drugs; This reuse of drugs is called drug repositioning (239). The use of drug repositioning can significantly reduce the cost and time of new treatments. A very indicative example of the application of drug repositioning was seen in the treatment of Covid-19. In addition to many computational and clinical studies, many drugs mentioned in computational studies could show appropriate effects in clinical studies. They were used as a treatment option in the treatment protocol for Covid-19 (240, 241).

Considering that no known drugs has yet been introduced as an effective intervention in the circadian cycle and approved in clinical trials, implementing drug repositioning to suggest effective medicinal compounds on different targets in the circadian cycle can be very practical and bring forth the treatment based on the circadian rhythm.

## 7 Conclusion

Various studies have shown the effect of circadian rhythm in the creation, progression, and treatment of diabetes, especially T2DM. These studies, which were mostly laboratory- and animal-based, have provided suitable evidence for designing and conducting further wider studies, especially at the clinical level. Moreover, chronotherapy has been proposed as one of the most trending areas of pharmacology, which is very important in making personalized medicine a reality in terms of more effectiveness and less side effects. Also, with a better understanding of chronotherapy, we can suggest combination treatments based on the combination of drugs with environmental and lifestyle factors. New laboratory tools and techniques such as organ-on-a-chip, microfluidic-based diagnostic tools, and new generation sequences allow us to conduct more studies on different aspects of the circadian rhythm and its relationship with diabetes. Also, with the progress of computational tools and biological modeling, as well as the huge growth of data science and artificial intelligence, chronotherapy appears to be a very promising approach in the treatment of different types of diseases.

## Author contributions

Conceptualization: MM, AH, and PS. Methodology: MM and AA. Investigation: AH, MK, MA-P, and AT. Resources: MM and AZ. Writing—original draft preparation: AH, MK, MA-P, AT, and PP. Writing—review and editing: MM, AZ, AA, and PS. All authors contributed to the article and approved the submitted version.
